# Ethnic and gender discrimination in the private rental housing market in Finland: A field experiment

**DOI:** 10.1371/journal.pone.0183344

**Published:** 2017-08-30

**Authors:** Annamaria Öblom, Jan Antfolk

**Affiliations:** Department of Psychology, Faculty of Arts, Psychology and Theology at Åbo Akademi University, Turku, Finland; University of Vermont, UNITED STATES

## Abstract

Ethnic and gender discrimination in a variety of markets has been documented in several populations. We conducted an online field experiment to examine ethnic and gender discrimination in the private rental housing market in Finland. We sent 1459 inquiries regarding 800 apartments. We compared responses to standardized apartment inquiries including fictive Arabic-sounding, Finnish-sounding or Swedish-sounding female or male names. We found evidence of discrimination against Arabic-sounding names and male names. Inquiries including Arabic-sounding male names had the lowest probability of receiving a response, receiving a response to about 16% of the inquiries made, while Finnish-sounding female names received a response to 42% of the inquires. We did not find any evidence of the landlord’s gender being associated with the discrimination pattern. The findings suggest that both ethnic and gender discrimination occur in the private rental housing market in Finland.

## Introduction

### Definition and types of discrimination

Discrimination towards different groups in our society is evidently occurring, although the expression of direct discrimination has been reduced in recent decades [1]. Differently from racial prejudice (attitudes), racial stereotypes (beliefs) and racism (ideologies), the term “discrimination” does not assume any underlying cause. [2]. Hence, an individual who has no intention to engage in discrimination may still treat individuals or groups unequally, based on their personal characteristics such as ethnicity and gender [3]. Forms of discrimination include *differential treatment* and *disparate impact*. Differential treatment occurs when individuals receive unequal treatment due to a personal characteristic. Disparate impact includes processes and decisions that may not have any discriminative content independently, yet produce or reinforce disadvantage towards one group over another [3]. Thus, being declined housing on the grounds of ethnicity is an example of differential treatment. Being declined housing on grounds that do not appear to be discriminatory may, however, be a case of disparate impact, if the act reinforces disadvantage over a minority group.

### The prevalence of discrimination

A review on racial discrimination in the US indicated the occurrence of discrimination in a variety of markets, such as employment, housing, credit, and consumer markets [3]. For example, a study showed that discrimination was perceived by 30% of the included minority-group in the US during 2008 [4]. Similar results have been found in studies conducted in Europe [5]. Discrimination in Europe is believed to be widespread, particularly on the grounds of age, gender and ethnicity [6]. The Eurobarometer 2015 showed an increase from 16% to 21% in the overall proportion of perceived discrimination in the European Union [7]. The rate of perceived discrimination in Finland in 2012 was 16% [8]. The most common bases for discrimination were ethnicity (69%) and gender (51%). Taken together, these results suggest that discrimination based on ethnicity and gender in a variety of countries continues to be prevalent.

### The association between discrimination and well-being

Previous research provides a clearly documented link between discrimination and psychological health [9–13]. In a meta-analysis, Schmitt, Branscombe, Postmes and Garcia showed a significant association between perceived ethnic discrimination and a decreased psychological well-being [14]. Further, results from a literature review indicated an association between that ethnic discrimination and psychological distress among immigrants [15]. Moreover, discrimination has been associated to an increase in anxious, depressive, psychosomatic, and physiological symptoms [1, 9, 16]. This is especially true in immigrant populations [1]. The clearly documented associations between discrimination and negative health effects make discrimination an important topic of research.

### Discrimination in the housing market

Housing-market discrimination occurs when individuals receive differential treatment based on personal factors such as ethnicity or gender [17]. The increased difficulties some groups face in gaining access to a suitable and well-located apartment is an example of such discrimination [18]. Previous research provides evidence of housing-market discrimination in countries such as the US, Australia, India, Canada, Germany, Spain and Sweden [3, 17, 19]. About 30% of the immigrants interviewed in a study conducted in Finland reported occurrence of discrimination in the apartment seeking process [18]. Compared to the ethnic majority, potential tenants belonging to an ethnic minority usually receive fewer responses to their inquiries on apartments and shown and offered fewer housing units, and may be invited to less apartment viewings [20]. A study from 2013 on housing policy in the Nordic countries showed that immigrants in Norway, Denmark, Sweden and Finland acquired different housing positions compared to the majority population [21].

Results from a recent field experiment on discrimination in the housing market in France revealed that applicants with foreign sounding names were less likely to receive responses to their e-mail enquiries than participants with French-sounding names [22]. In addition, applicants with foreign-sounding names received responses that were more likely to be negative. Similar results were found in a recent study in Belgium [23]. A study on rental discrimination and ethnicity in the US showed that typical African-American and Arabic names received fewer positive responses than typical White names [24]. In sum, results from previous studies suggest that ethnic discrimination in the housing market occurs in a variety of countries, and that discrimination is based on different response patterns to inquiries made with different names.

Gender discrimination is also frequently associated with ethnic discrimination in housing market studies [20, 25, 26]. A field experiment on gender and ethnic discrimination in the Norwegian housing market showed that Arabic males in particular were discriminated. Females with an Arabic name were not discriminated against to the same degree [25]. Similar results were found in a Swedish field experiment [20]. Females with a Swedish name received more responses to their apartment inquiries than males with a Swedish name, and males with an Arabic name received worse treatment than males with a Swedish name. However, Bengtsson and colleagues extended the study and found that females with a Swedish name were more likely to receive responses from landlords than females with Arabic names indicating an ethnic discrimination also amongst females [26]. Together, previous studies indicate that females with native names are more likely to receive responses than females with foreign names when applying for an apartment, and males with foreign names may face more discrimination than both females with foreign names and males with native names.

### The housing market in Finland

During 2015, 1.4 million of the Finnish population lived in rental apartments and the share of rental apartments in the housing market was approximately 32% [27]. The size-corrected price (€/m^2^) for rental apartments in three large cities in Finland during 2016 was 16.14€/m^2^ in Helsinki, 13.22€/m^2^ in Tampere, and 12.37€/m^2^ in Turku. The mean apartment price in the whole country was 12.72€/m^2^ [27]. The capital area of Helsinki is the most expensive area to live in, and it can be difficult to gain access to apartments [28]. In Finland an act of Non Discrimination from 2014 is adapted on all housing policies [29]. It states that no one may be discriminated against on the basis of personal characteristics, and landlords are obliged to follow this legislation when renting an apartment [30]. The act of Non Discrimination also applies to online rental housing, that has become a widely used platform for apartment distribution in Finland [31, 32].

### The current study

Discrimination can be examined with surveys and self-reports (perceived discrimination and attitudes), statistical analyses of demographic data, and laboratory experiments [33]. Laboratory experiments often offer the strongest evidence of causality, but might in turn have low ecological validity [34]. To address the issue of ecological validity, scholars have used field experiments such as *audit tests* or *correspondence tests* [34]. Audit tests involve applicants of different ethnicity (matched on variables like gender, education and physical appearance) who apply for an apartment or a job in person. Discrimination is measured as the number of responses or offers an applicant receives. However, tests based on personal approaches are not double-blind, and traits unaccounted for in the audit design could lead to bias in either direction [35–37]. Hence, many scholars have chosen to use correspondence tests rather than audit tests [38].

In the current study, we examined discrimination by using a correspondence test. We made this choice because correspondence tests do not involve real-life contact between a tester or confederate and the test case. Instead, we sent written applications to landlords, which also mimics the most prevalent form of contact between landlords and possible tenants. The application process is terminated when the applicant receives an answer to their inquiry. Correspondence testing allows for control of the contents of the application, and for randomization of the applicant names. In addition, correspondence testing is cost-effective, and enables the use of large sample sizes [38]. Further, correspondence tests are less subjective than audit tests, as researchers or assistants are less likely to involuntarily influence landlords’ decision-making processes.

Our intention was to examine the effects of ethnicity and gender on the probability to receive a response from a landlord to an apartment inquiry. We were interested in whether females and males with Arabic-sounding names were more likely to experience discrimination than females and males with Finnish-sounding or Swedish-sounding names. Immigrants born in Somali and Iraq belong to two the largest immigrant groups in Finland [9, 39], and therefore the possible discrimination of individuals with Arabic-sounding names is of particular interest. Swedish-sounding names were included because Finland is a bilingual country, with Finnish and Swedish as the two main languages. By the end of 2013, 5.3% of the Finnish population was Swedish-speaking [40]. According to research on cultural differences between the two language groups, the Swedish-speaking Finns have slightly better health, employment, income, and equal to higher social status than Finnish-speaking Finns [41, 42]. We were therefore also interested in exploring landlords’ responses to individuals with Finnish-sounding and Swedish-sounding names.

In the current study, we sent out standardized apartment inquiries to 800 landlords on the online platform Tori.fi. The inquiry contained a request for more information about the apartment. We used fictive names that indicated ethnicity and gender of the applicant. We randomized the selection of a name to be included in an inquiry. We then analyzed the received responses, which served as measures of discrimination.

Based on the aforementioned studies in the field, we formulated the following hypotheses:

1. Ethnicity-based discrimination, such that Arabic-sounding names would receive less favorable responses than Finnish and Swedish names;
2. Gender-based discrimination, such that male names would receive less favorable responses than female names; and
3. An interaction effect of the two types of discrimination, such that the gender discrimination (discrimination of men) would be larger for Arabic-sounding names than for Finnish-sounding and Swedish-sounding names.

## Materials and methods

### Ethics statement

The current study received permission by the Faculty of Arts, Psychology and Theology at Åbo Akademi University, and by the Ethical board of Åbo Akademi University during fall 2015.

### Procedure

To investigate discrimination in the private housing market we used Tori.fi. Tori.fi is a large online platform in Finland used for renting apartments. In this procedure, we complied with the terms and conditions for the online platform. We conducted the study between December 2015 and April 2016. Included in the study were three metropolitan areas in Finland: Helsinki, Turku and Tampere. In addition, we added the group “Others” that consisted of apartment advertisements from randomly selected locations around Finland. We replied to housing advertisements posted online during the prior month, and included advertisements for any type of housing. Eight-hundred selected advertisements were included in the sample. We excluded advertisements looking for an applicant of a specific gender.

We created six fictitious e-mail accounts based on fabricated names on the Gmail platform. Each account represented the ethnic background and gender of an applicant applying for housing online. We included names that represented regular names of individuals belonging to three different ethnic groups in Finland. We included Arabic-sounding names, Finnish-sounding names and Swedish-sounding names. For the Finnish-sounding and the Swedish-sounding names, frequency data from the Population Register Centre in Finland was used [43, 44]. See S1 Appendix for the names used in this study.

For each apartment advertisement, we randomly selected a pair of names, and sent two matched inquiries to the landlord of the included advertisement. This was done by randomly drawing a number between 1 and 6 (each number corresponding to one of the names) for the first inquiry. We also drew a number between 1 and 6 for the second inquiry. In the cases both randomizations produced the same number, the randomization of the second inquiry was repeated until a different number was produced. Each inquiry was identical and written in grammatically sound Finnish. See S1 Appendix for an example. The second inquiry was sent one to three days after the first one. Our aim was to send 1600 inquiries in total. In 141 cases the apartment advertisement had been removed after the first inquiry was sent, and thus only one inquiry per landlord was sent in these instances. This resulted in 1459 sent inquiries in total. To comply with the directives defined in the ethical approval we sent debriefing e-mails to the participants during the month of January 2017. To ensure confidentiality of the participants, data that potentially could reveal the identify (e-mail addresses needed for the debriefing, address of the apartment, and dates and contents of e-mails) were stored in a data file only accessible to the first author. After coding landlords’ gender, names were also removed from this file. A public file data without any identifying information is stored at the Open Science Framework.

### Measures

To operationalize discrimination, we used two different indicators. Firstly, we registered whether each inquiry received an answer or not within the time frame of the study. Secondly, received responses were coded as either *positive* (indicating an invitation to further contact with the landlord or receiving an invitation to a viewing) or as *negative* (indicating a rejection of further contact with the landlord or receiving a response about an unavailable apartment). In our study, we also calculated the ratio of positive responses (positive responses / all inquiries). Additionally, we coded gender of the landlord using binary coding (1 for male and 2 for female). When the gender could not be reliable obtained, we treated gender information as missing. We also registered the location, size and prize of the housing.

The coding of responses as positive or negative was done by one of the authors, and a randomly selected subset of 30 responses were selected for assessment of coding agreement. The other author coded these responses independently. There was a 100% agreement between the two coders (κ = 1.00).

### Data analysis

Because observations were clustered within individual landlords, we conducted our main analyses using a generalized linear mixed model in the *lme4*-package [45] for R [46]. To model data we used binomial logistic regressions fit by a maximum likelihood estimation (Laplace approximation). In the first regression model, the probability of receiving a response was the criterion. In the second regression model, the probability of receiving a positive response was the criterion. In both models, we investigated both the main effects of ethnicity and gender as well as their interaction. Thus, gender and ethnicity were treated as independent factorial predictors.

For example, we specified the full factorial binomial logistic regression model for the probability of receiving a positive response as follows:

Model <− glmer(Response ~ Ethnicity + Gender + Gender * Ethnicity + (1 | Landlord), family = “binomial”)

We also generated mixed model ANOVA tables using the *afex*-package [47]. For the main and interaction effects, p-values were calculated using the documented likelihood-ratio test. We used one-sided Tukey tests in the *multcomp*-package for post-hoc comparisons between levels [48]. To obtain point estimates and *SE* that from the mixed model, we added the term “-1” after the predictor term. This removes the intercept from the model and the yields absolute estimates for each included condition. The point estimates (log odds ratios) for each condition were then converted into probabilities using the formula

$$\frac{\text{exp}\left(x\right)}{1+\text{exp}\left(x\right)}$$

where x is the point estimate of interest. The 95%CI were obtained through a similar conversion after multiplying the *SE* by 1.96 and subtracted from (for the lower bound estimates) or added to (for upper bound estimates) the point estimate. Correlations of fixed effects are reported in the S1 and S2 Tables.

As explorative analyses, we also split the data according to the landlord gender and ran similar analyses for both subgroups of male and female landlords.

χ²-test and one-way ANOVAs with Tukey HSD pairwise comparison test were used to examine descriptive data.

## Results

### Descriptive statistics

Out of the 1459 inquires, 722 included a male name and 737 included a female name. Four-hundred and ninety-four inquires included an Arabic-sounding name, 488 a Swedish-sounding name, and 477 a Finnish-sounding name. The distribution of ethnicities was similar for both genders. The main descriptive statistics, distributions of sent inquiries and received responses are shown in Table 1. To test whether our sampling reflected the general housing market in Finland, we also calculated the mean and *SD* for the price (€/m^2^) for each measured location (Helsinki, Turku, Tampere and Other). We found that the distribution of prices reflected those provided from Statistics Finland [27]. In all cases, the price (€/m^2^) provided by Statistics Finland was within 1 *SD* of our mean. See S3 Table for more details.

**Table 1:** Distribution of sent e-mails and received responses from all landlords, female landlords and male landlords.

	Arabic		Finnish		Swedish		Landlords			Total
	Female	Male	Female	Male	Female	Male	Female (*N* = 350)	Male (*N* = 411)	Unknown (*N* = 39)	(*N* = 800)
Sent e-mails	249	245	235	242	253	235	643	746	70	1459
Received response										
Yes	79	44	100	84	96	91	216	258	20	493
No	170	201	135	158	157	144	426	489	50	966
Response type										
Positive	66	35	85	76	89	80	192	223	16	431
Negative	13	9	15	8	7	11	24	35	4	63

### Response and a positive response by ethnicity and gender

We first investigated the main effects of gender and ethnicity on the probability to receive a response to an apartment inquiry and on the probability to receive a positive response. In the same model (one per each dependent variable), the interaction term was also included. The main effects of gender and ethnicity were statistically significant, and so was the interaction effects, see Table 2.

**Table 2:** ANOVA table for mixed model effects.

	Response			Positive response		
	*df*	χ^2^	*p*	*df*	χ^2^	*p*
Gender	1	10.11	< .01	1	8.93	< .01
Ethnicity	2	31.27	< .001	2	35.21	< .001
Gender x Ethnicity	2	7.81	< .05	2	6.32	< .05

Concerning our hypothesis (1) that there would be a main effect of ethnicity on the probability to receive a response to an apartment inquiry, responses were significantly less likely for Arabic-sounding names than for Finnish-sounding names (-0.50, *SE* = 0.20, *z* = 2.46, *p* < .05), but not Swedish-sounding names (-0.31, *SE* = 0.20, *z* = 1.56, *p* = .13). There was no statistically significant difference between Finnish-sounding and Swedish-sounding names (0.19, *SE* = 0.20, *z* = 0.94, *p* = .31). Positive responses were significantly less likely for Arabic-sounding names compared to both Swedish-sounding (-0.49, *SE* = 0.22, *z* = 2.28, *p* < .05 and Finnish sounding names (-0.47, *SE* = 0.22, *z* = 2.18, *p* < .05). Again, there was no statistically significant difference between Finnish-sounding and Swedish-sounding names (0.02, *SE* = 0.21, *z* = 0.12, *p* = .45)

Concerning our hypothesis (2) that there would be a main effect of gender on the probability to receive a response to an apartment inquiry, responses were less significantly likely when inquiries were made using male names compared to female names (-0.82, *SE* = 0.23, *z* = 3.58, *p* < .001). Also positive responses were less likely when inquiries were made using male names compared to female names (= -0.86, *SE* = 0.25, *z* = 3.42, *p* < .001).

**Fig 1:**
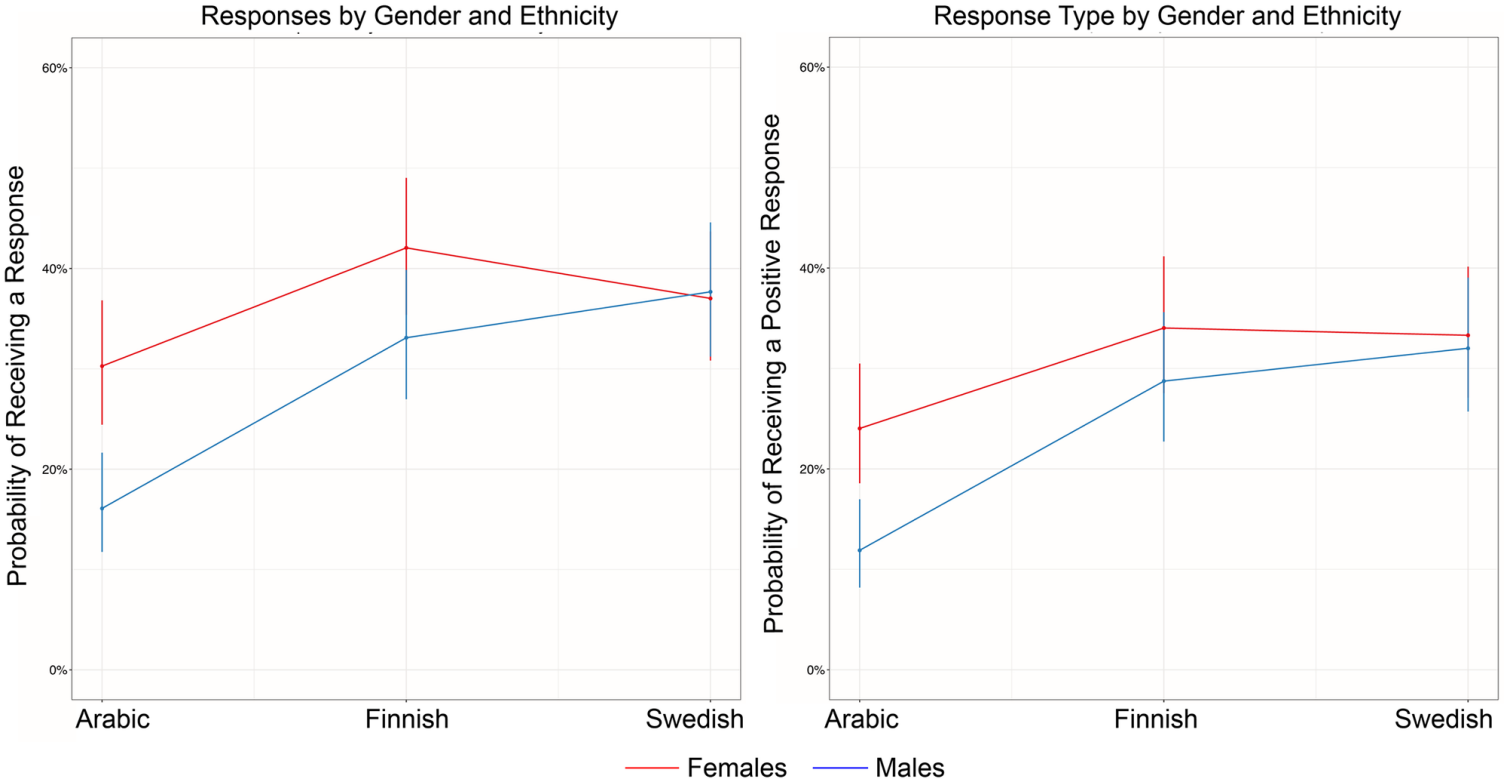
Responses and positive responses by gender and ethnicity. Predicted probabilities for response (y-axis; left panel) and predicted probabilities for positive response (y-axis; right panel) by gender (Female and Male) and ethnicity (Arabic, Finnish, and Swedish). Vertical lines denote the 95% confidence intervals.

Concerning our hypothesis (3) on an interaction effect of the two types of discrimination, responses where significantly less likely when inquiries were made using Arabic-sounding male names compared to all other names (all *p* <. 01). This was also true for positive responses. See Fig 1.

### Responses and a positive response by ethnicity and gender: Female and male landlords

To explore the association between discrimination based on tenants’ ethnicity and gender and landlord gender, we included landlord gender in the model. We found no evidence of a three-way interaction between landlord gender and tenant gender and ethnicity (χ^2^ [2] = 0.90, *p* = .64 for responses and χ^2^ [2] = 0.34, *p* = .84 for positive responses). Neither did we find any two-way interactions between landlord gender and tenant gender (χ^2^ [1] = 2.16, *p* = .14 for responses and χ^2^ [1] = 2.27, *p* = .13 for positive responses) or between landlord gender and landlord ethnicity (χ^2^ [2] = 2.96, *p* = .23 for responses and χ^2^ [2] = 3.80, *p* = .15 for positive responses). There was also no direct association between landlord gender (χ^2^ [1] = 0.51, *p* = .47 for responses and χ^2^ [1] = 0.21, *p* = .65 for positive responses). Thus, we did not find any evidence of the landlord’s gender being associated with discrimination. See Table 3 for probabilities for male and female landlords separately.

**Table 3:** Probabilities (and corresponding 95%CI) to receive a response and a positive response from all landlords, male landlords and female landlords.

Condition	Response						Positive response					
	All landlords		Male landlords		Female landlords		All landlords		Male landlords		Female landlords	
	Prob	95%CI [LL, UL]	Prob	95%CI [LL, UL]	Prob	95%CI [LL, UL]	Prob	95%CI [LL, UL]	Prob	95%CI [LL, UL]	Prob	95%CI [LL, UL]
Arabic												
Female	0.30	[0.24, 0.37]	0.34	[0.25, 0.43]	0.26	[0.18, 0.36]	0.24	[0.19, 0.30]	0.28	[0.23, 0.38]	0.22	[0.17, 0.31]
Male	0.16	[0.12, 0.22]	0.20	[0.13, 0.28]	0.14	[0.08, 0.23]	0.12	[0.09, 0.17]	0.15	[0.11, 0.23]	0.10	[0.07, 0.17]
Finnish												
Female	0.42	[0.35, 0.49]	0.38	[0.30, 0.44]	0.45	[0.35, 0.55]	0.34	[0.28, 0.41]	0.30	[0.24, 0.40]	0.39	[0.33, 0.50]
Male	0.33	[0.27, 0.40]	0.34	[0.26, 0.44]	0.32	[0.23, 0.43]	0.29	[0.23, 0.36]	0.30	[0.27, 0.44]	0.28	[0.22, 0.39]
Swedish												
Female	0.37	[0.31, 0.44]	0.34	[0.25, 0.44]	0.43	[0.34, 0.53]	0.34	[0.28, 0.40]	0.30	[0.24, 0.39]	0.39	[0.33, 0.49]
Male	0.38	[0.31, 0.45]	0.41	[0.31, 0.51]	0.34	[0.26, 0.44]	0.32	[0.26, 0.39]	0.33	[0.27, 0.44]	0.30	[0.24, 0.40]

CI = Confidence Interval; LL = Lower Level; UL = Upper Level.

## Discussion

The aim of the present field experiment was to examine whether or not and to which extent, ethnic and gender discrimination occur in the private rental housing market in Finland. We conducted the study using a correspondence test in which we sampled 800 rental advertisements from a market-leading online site. We sent inquires that included either male or female Arabic-, Finnish- or Swedish-sounding names. Hence, in the current study we focused on discrimination as behavior, measured as responses to apartment inquiries.

In line with previous research we found discrimination against Arabic-sounding names [20, 24]. Inquires including Arabic-sounding names had a markedly lower probability of receiving a response than Finnish- and Swedish-sounding names. We also found that inquires including female names were more likely to receive responses than males. Moreover, the discrimination against men was largest for inquires including Arabic-sounding names. In fact, there was no evidence of an effect of gender for inquires including Finnish- or Swedish-sounding names. In addition to the aforementioned results, we also compared received responses between female and male landlords but did not find any significant differences. To our best knowledge, this was the first correspondence study of discrimination in the Finnish housing market.

Our results suggest the occurrence of discrimination in the private housing market in Finland. This discrimination most clearly has a negative effect for Arabic males. Also Arabic females are less likely to gain access to the rental market, compared to females with Finnish- or Swedish-sounding names. This is in line with earlier research, showing that an individual belonging to an ethnic minority usually has a lower probability of receiving a response to an apartment inquiry [20–24].

Our results also support findings from former studies conducted in Finland and other Nordic countries [20, 21]. Results from Finland, Sweden, Norway and Denmark suggest the occurrence of both ethnic and gender discrimination in housing markets. However, unlike in the study by Bengtsson and colleagues in Sweden, discrimination amongst females with Arabic-sounding names was not prevalent [26].

### Implications

Access to housing is of paramount importance for functioning and well-being in other areas of life. It affects health, personal and professional opportunities, and the availability of public services and social networks [19, 49]. It is therefore important that individuals have equal, unbiased access to the housing market. Previous research has investigated the sequelae of housing discrimination and shown a variety of adverse economic and social consequences for discriminated groups. These sequelae include worsening residential segregation, poorer educational access, poorer employment rates, and a decreased welfare for members of the discriminated groups [19, 26, 49]. Thus, promoting ethnic and gender equality in the rental housing market may have several positive long-term consequences for the individual, as well as positive social, economic, and public health consequences for society.

The implications of our results are straightforward. Individuals with Arabic-sounding male names, and to lesser extent individuals with Arabic-sounding female names, may find it more difficult to acquire rental housing. This might affect their well-being on many levels, as indicated by prior research [9–13]. Demonstrating the occurrence of ethnic and gender discrimination in the Finnish housing market is also useful for policymaking. Both forms of discrimination can be either intentional or unintentional, and thus, cases of differential treatment or disparate impact [2, 3]. The act of Non Discrimination from 2014 states that no one may be discriminated against based on personal characteristics such as nationality and gender [29]. This act should be adapted to all housing policies, including the online platform for rental apartments. However, based on the results of this study, the Non Discrimination act may not prevent the occurrence of discrimination (against Arabic-sounding names and male names) in the housing market. Thus, our findings provide relevant information for the future development of non-discrimination and equal housing opportunities in the private housing market in Finland.

### Limitations and future directions

A limitation of the present field experiment is that it investigated only differences in housing access between individuals with Finnish-, Swedish- and Arabic-sounding names. We did not use names of non-Arabic immigrants, and it is therefore currently impossible to generalize the observed effect to other immigrant groups. Moreover, a limited number of names were included in the study, and we chose to include names that could be considered stereotypical. Some less typical ethnic names may be misattributed to other ethnic groups, and thus, stereotypical ethnic names may lead to different responses than less stereotypical names [50]. These issues are worth consideration in future studies.

The validity of response rates as a measure of discrimination is also not perfect as there can be several different reasons for not replying to an inquiry [5]. However, due to the experimental design including random allocation of names to inquiries for apartments it is unlikely that any systematic bias would be confounding the results of the current study. It is important to keep in mind that the response rates do not reflect the probability of receiving a response if replying to a recently added advertisement. Because we sent inquiries to advertisements published within the last month, some of these might already have been rented (but not removed from the sites). Although this decreases the overall probability of receiving a response, it did not affect the estimated discrimination as we used a randomized procedure in the current study.

It is likely that some of the landlords were couples and that, in many cases, the decision regarding whether to respond was a decision made by both a male and female landlord. This naturally decreases the validity of the gender coding for landlords, and obscures analyses including landlord gender. It is also worth noting, that landlord gender was not experimentally manipulated. Because of this, no causal interpretations of can be made.

An additional limitation to the current study is that a correspondence test does not capture the underlying attitudes and perceptions that may be associated with the very acts of discrimination [3, 34, 51]. It is therefore impossible to draw any conclusions regarding the motives or individual propensities that lead to the observed discriminatory behavior.

A suggestion for future research is to compare responses to apartment inquiries between different metropolitan areas in Finland. This would allow for an investigation of whether the high demand and the expensive apartment prices in Helsinki are associated to higher rates of discrimination. Although the current study included all the necessary variables for such an analyses, the subsample for a specific region was too small to conduct meaningful statistical analyses.

### Conclusions

Taken together, the results from the current study support findings from previous studies on ethnic and gender discrimination in housing markets of several nations. We provide evidence of the occurrence of both ethnic and gender discrimination in the Finnish private rental housing market, such that individuals with Arabic-sounding names and individuals with male names are discriminated. Males with Arabic-sounding names have the lowest probability to receive a response to an apartment inquiry. We do not find any evidence of the landlord’s gender affecting discrimination. This study contributes by providing valuable knowledge to the area of discrimination research in the housing market in Finland.

## Supporting information

S1 Appendix Applicant names. Inquiry sent to landlords.
(PDF)

S1 Table Correlation of fixed effects on the probability to receive a response. (PDF)

S2 Table Correlation of fixed effects on the probability to receive a positive response. (PDF)

S3 Table Apartment prices per location. (PDF)
