# Supplementary material for: Ethnic and gender discrimination in the private rental housing market in Finland: A field experiment
Source: PLoS One. 2017 Aug 30;12(8):e0183344. doi: 10.1371/journal.pone.0183344 (PMC5576692; doi:10.1371/journal.pone.0183344)
Supplement: S1 Appendix — Inquiry sent to landlords. (PDF) [file pone.0183344.s001.pdf]

S1 Appendix

Applicant names

1. Juuso Laine (Finnish male)
2. Anu Koskinen (Finnish female)
3. Erik Johansson (Swedish male)
4. Elisabeth Andersson (Swedish female)
5. Ali Hussein (Arabic male)
6. Miriam Al-Zahavi (Arabic female)

Inquiry sent to landlords

Original

Hei,
olen kiinnostunut ilmoittamastasi asunnosta, Olisiko asunnosta mahdollista saada lisäinfoa sähköpostitse?
Ystävällisin terveisin,
Xx

In English

Hello,
I am interested in the apartment you advertised. Is it possible to get some further information of the apartment per e-mail?
Kind regards,
Xx
