# Supplementary material for: Ethnic and gender discrimination in the private rental housing market in Finland: A field experiment
Source: PLoS One. 2017 Aug 30;12(8):e0183344. doi: 10.1371/journal.pone.0183344 (PMC5576692; doi:10.1371/journal.pone.0183344)
Supplement: S1 Table — (PDF) [file pone.0183344.s002.pdf]

S1 Table. Correlation of Fixed Effects on the Probability to Receive a Response.

All landlords[a]		Intercept	GndrMl	EthncS	EthncF	GnM:ES
	GenderMale	-0.546				
	EthnicitySwe	-0.719	0.439			
	EthnicityFinn	-0.702	0.439	0.532		
	GndrMl:EthS	0.425	-0.765	-0.641	-0,339	
	GndrMl:EthF	0.426	-0.757	-0.338	-0.653	0.579
Male landlords [b]		GndrFm	GndrMl	EthncS	EthncF	GnM:ES
	GenderMale	0.130				
	EthnicitySwe	-0.683	-0.025			
	EthnicityFinn	-0.679	-0.010	0.497		
	GndrMl:EthS	0.421	-0.538	-0.665	-0.333	
	GndrMl:EthF	0.424	-0.549	-0.328	-0.673	0.543
Female landlords [c]		GndrFm	GndrMl	EthncS	EthncF	GnM:ES
	GenderMale					
	GenderMale	0.134				
	EthnicitySwe	-0.761	-0.064			
	EthnicityFinn	-0.728	-0.047	0.573		
	GndrMl:EthS	0.435	-0.607	-0.612	-0.347	
	GndrMl:EthF	0.429	-0.575	-0.345	-0.623	0.614

Fm = female; Ml = male; ES = Ethnicity Swedish.

[a] glme: intercept “Arabic Female

[b]glme: one variable with six levels

[c]glme: one variable with six levels
