# Supplementary material for: Ethnic and gender discrimination in the private rental housing market in Finland: A field experiment
Source: PLoS One. 2017 Aug 30;12(8):e0183344. doi: 10.1371/journal.pone.0183344 (PMC5576692; doi:10.1371/journal.pone.0183344)
Supplement: S2 Table — (PDF) [file pone.0183344.s003.pdf]

S2 Table. Correlation of Fixed Effects on the Probability to Receive a Positive Response.

All landlords [a]		Intercept	GndrMl	EthncS	EthncF	GnM:ES
	GenderMale	-0.585				
	EthnicitySwe	-0.717	0.444			
	EthnicityFinn	-0.710	0.441	0.520		
	GndrMl: EthS	0.444	-0.752	-0.652	-0.334	
	GndrMl: EthF	0.450	-0.744	-0.337	-0.660	-0.559
Male landlords[b]		GndrFm	GndrMl	EthncS	EthncF	GnM:ES
	GenderMale	0.082				
	EthnicitySwe	-0.689	-0.023			
	EthnicityFinn	-0.700	-0.019	0.496		
	GndrMl: EthS	0.440	-0.534	-0.672	-0.331	
	GndrMl: EthF	0.454	-0.538	-0.330	-0.676	0.531
Female landlords[c]		GndrFm	GndrMl	EthncS	EthnF	GnM:ES
	GenderMale	0.088				
	EthnicitySwe	-0.757	-0.047			
	EthnicityFinn	-0.730	-0.039	0.560		
	GndrMl:EthS	0.460	-0.590	-0.633	-0.350	
	GndrMl:EthF	0.449	-0.561	-0.348	-0.638	0.588

Fm = female; Ml = male; ES = Ethnicity Swedish.

[a] glme: intercept “Arabic Female”

[b]glme: one variable with six levels

[c]glme: one variable with six levels
