# Supplementary material for: Ethnic and gender discrimination in the private rental housing market in Finland: A field experiment
Source: PLoS One. 2017 Aug 30;12(8):e0183344. doi: 10.1371/journal.pone.0183344 (PMC5576692; doi:10.1371/journal.pone.0183344)
Supplement: S3 Table — (PDF) [file pone.0183344.s004.pdf]

S3 Table. Apartment Prices per Location.

Location	*n*	Mean apartment prize in €/m²	*SD*	Pairwise comparisons	
Helsinki	193	21.02	7.19	Turku:	-6.70***
				Tampere:	-5.64***
				Other:	-9.54***
Turku	193	14.32	4.25	Tampere:	1.06
				Other:	-2.83***
Tampere	192	15.38	4.82	Other:	-3.89***
Other	190	11.49	3.46		

*** $p < .001$, ** $p < .01$, * $p < .05$. *n* = number of apartments by location for which price and size information was available. *p*-values for pairwise comparisons are based on a Tukey HSD post hoc pairwise comparison. A One-way ANOVA (F [3] = 116.6, $p < .001$) showed statistically significant differences between rental prices for the included areas. Prices significantly differ between all the other areas included, but Turku and Tampere.
